# Precision Phage Cocktail Targeting Surface Appendages for Biocontrol of *Salmonella* in Cold-Stored Foods

**DOI:** 10.3390/antibiotics13090799

**Published:** 2024-08-24

**Authors:** Seongok Kim, Bokyung Son, Hyeryen Kim, Hakdong Shin, Sangryeol Ryu

**Affiliations:** 1 Department of Food Science & Biotechnology, College of Life Science, Sejong University, Seoul 05006, Republic of Korea; skim01@sejong.ac.kr; 2 Carbohydrate Bioproduct Research Center, College of Life Science, Sejong University, Seoul 05006, Republic of Korea; 3 Department of Food Biotechnology, Dong-A University, Busan 49315, Republic of Korea; bkson@dau.ac.kr; 4 Department of Food and Animal Biotechnology, Research Institute of Agriculture and Life Sciences, Seoul National University, Seoul 08826, Republic of Korea; luckposi@gmail.com; 5 Department of Agricultural Biotechnology, Seoul National University, Seoul 08826, Republic of Korea; 6 Center for Food and Bioconvergence, Seoul National University, Seoul 08826, Republic of Korea

**Keywords:** phage cocktail, *Salmonella*, biocontrol, cold storage

## Abstract

*Salmonella enterica* is a major food-borne pathogen causing food poisoning. The use of bacteriophages as alternative biocontrol agents has gained renewed interest due to the rising issue of antibiotic-resistant bacteria. We isolated and characterized three phages targeting *Salmonella*: SPN3US, SPN3UB, and SPN10H. Morphological and genomic analyses revealed that they belong to the class *Caudoviricetes*. SPN3UB, SPN3US, and SPN10H specifically target bacterial surface molecules as receptors, including O-antigens of lipopolysaccharides, flagella, and BtuB, respectively. The phages exhibited a broad host range against *Salmonella* strains, highlighting their potential for use in a phage cocktail. Bacterial challenge assays demonstrated significant lytic activity of the phage cocktail consisting of the three phages against *S. typhimurium* UK1, effectively delaying the emergence of phage-resistant bacteria. The phage cocktail effectively reduced *Salmonella* contamination in foods, including milk and pork and chicken meats, during cold storage. These results indicate that a phage cocktail targeting different host receptors could serve as a promising antimicrobial strategy to control *Salmonella*.

## 1. Introduction

*Salmonella* is a leading cause of foodborne illness, responsible for 1.35 million infections, 26,500 hospitalizations, and 420 deaths in the United States every year [1]. According to the Interagency Food Safety Analytics Collaboration, over 23% of foodborne *Salmonella* illnesses are associated with the consumption of poultry meat [2]. *Salmonella* has more than 2,600 different serotypes identified, of which *Salmonella typhimurium* and *Salmonella enteritidis* are the two major causes of foodborne illnesses [3,4]. These species are transmitted from humans, leading to severe gastrointestinal diseases, characterized by symptoms such as diarrhea, fever, and abdominal cramps [5]. Unfortunately, controlling *Salmonella* has become increasingly challenging due to the development of strains resistant to multiple antibiotics [6,7]. The emergence and widespread prevalence of antibiotic-resistant bacteria pose a significant global public health threat, necessitating the exploration of alternative antibacterial strategies.

In recent years, the application of phages, viruses that specifically infect and cause bacterial lysis, has attracted significant attention as an alternative to antibiotics because of their host specificity, effective host–cell lysis, and safety to humans [8,9]. In particular, phages with a lytic life cycle have been researched for various applications in the food industry [10]: the prevention of pathogen colonization in livestock, decontamination of carcasses and other ready-to-eat (RTE) foods, disinfection of pathogen contact surfaces, and preservation of foods [11]. A phage cocktail composed of phages with different infection strategies has also been employed to enhance host growth inhibition as it can broaden the host range and reduces the chance of resistance development [12,13]. Recent studies have focused on using a phage cocktail to control *S. typhimurium* in foods, providing several promising outcomes. For example, Abhisingha et al. investigated the effectiveness of a phage cocktail containing two *Salmonella* phages, ENT101 and TYM101, in controlling *S. typhimurium* on chicken meat [14]. This phage cocktail achieved a 0.4–1 log CFU/cm^2^ reduction in *Salmonella* quantity. The research also involved the evaluation of the antibacterial activity of phage cocktails at different temperatures, demonstrating the potential of phages in reducing *Salmonella* contamination in poultry products under various conditions. Another novel phage cocktail consisting of three phages (BSPM4, BSP101, and BSP22A) that target different host receptors was developed [15]. The phage cocktail not only delayed the emergence of *Salmonella* resistance but also significantly reduced viable *Salmonella* cell numbers in fresh produce. These findings encourage the development of new strategies using a phage cocktail to reduce the incidence of foodborne illnesses caused by *Salmonella* contamination.

In this study, we isolated and characterized three phages targeting *Salmonella*—SPN3US, SPN3UB, and SPN10H—by analyzing their morphology, host range, bacterial growth inhibition, and genomic characteristics. A phage cocktail composed of the three phages was effective in killing *S. typhimurium* on various food matrices at a refrigerated temperature and delayed the emergence of resistance.

## 2. Results and Discussion

### 2.1. Morphological and Genomic Features of Phages

**Figure 1:**
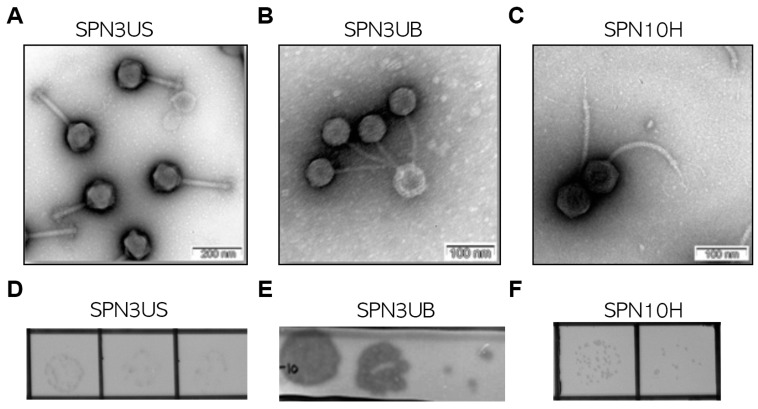
Morphological features of phages. TEM image of phage SPN3US (**A**), SPN3UB (**B**), and SPN10H (**C**) with head/tail structure. Scale bar: 200 nm (**A**) and 100 nm (**B**,**C**). Images of plaques formed by phage SPN3US (**D**), SPN3UB (**E**), and SPN10H (**F**), respectively.

To characterize the morphology of three isolated phages, Transmission Electron Microscopy (TEM) analysis was performed. The analysis revealed that the phages belong to the class *Caudoviricetes* but each phage has distinct morphological characteristics [16]. The result revealed that SPN3US had a non-flexible and contractile tail (200 ± 22 nm) with an icosahedral head (110 ± 9 nm), indicative of a myovius-like morphology (Figure 1A). In contrast, both SPN3UB and SPN10H exhibited smaller heads with a diameter of 55 ± 3 nm and 65 ± 5 nm, respectively, and flexible tails with a length of 156 ± 11 nm and 220 ± 12, respectively (Figure 1B, C). These features suggest SPN3UB and SPN10H have a sipovius-like morphology (Figure 1B, C). According to the International Committee on Taxonomy of Viruses (ICTV, https://ictv.global/, accessed on 20 August 2024), SPN3US belongs to the *Seoulvirus* genus of the *Chimalliviridae* family and SPN3UB shares genetic homologies with phages in the *Lederbergvirus* genus. SPN10H belongs to the *Demerecviridae* family, genus *Epseptimavirus*.

**Figure 2:**
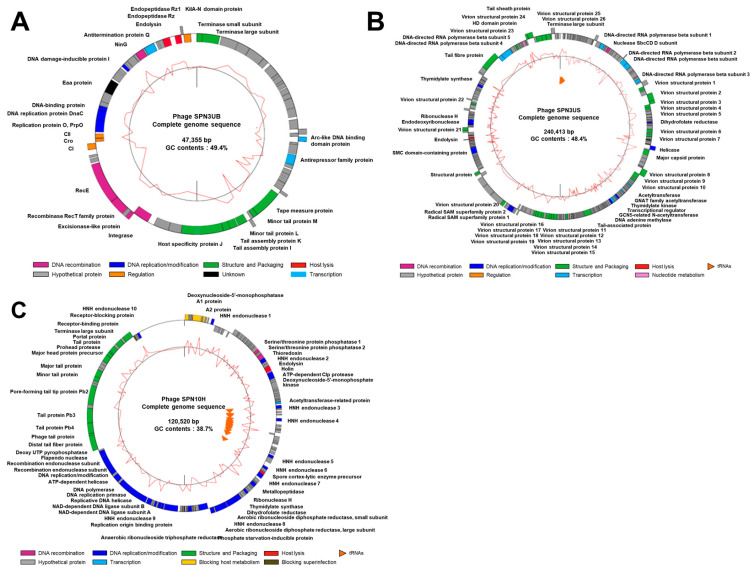
Complete genome maps of (**A**) SPN3UB, (**B**) SPN3US, and (**C**) SPN10H. The outer histogram map indicates gene coding regions by strands. The color of each gene represents the functional group: DNA recombination (magenta), DNA replication/modification (blue), nucleotide metabolism (pink), structure and packaging (green), host lysis (red), regulation (orange), unknown function (black), transcription (sky blue), blocking host metabolism (yellow), blocking superinfection (brown), and hypothetical protein (gray). The inner circles with the red line indicate the GC contents, and the orange arrowheads represent the location of tRNAs. Genome maps were generated using DNASTAR GeneScene v.0.99.8.0 (dnastar.com).

The SPN3UB genome encodes phage structural proteins, phage packaging terminases, lysogeny control proteins, phage replication proteins, host–cell lysis enzymes and peptidases, and proteins with various other functions (Figure 2A; [17]). SPN3US genome comprises functional genes associated with phage structure and packaging, tail structure, replication/transcription, host lysis and additional accessary proteins (Figure 2B; [18]). The SPN10H genome includes functional genes responsible for head/tail structure, phage replication, and host–cell lysis enzymes and peptidases (Figure 2C).

**Figure 3:**
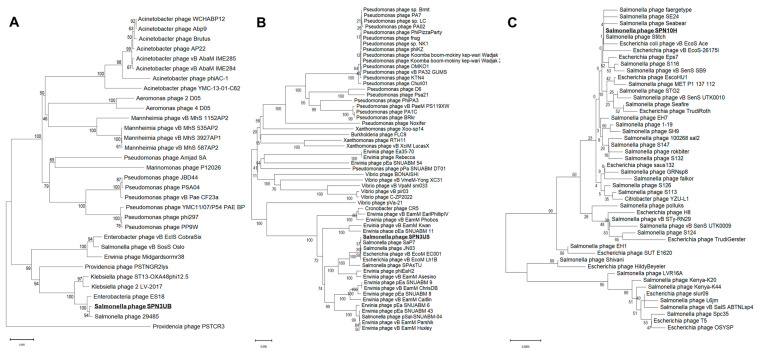
Neighbor-joining phylogenetic trees of phage SPN3UB (**A**), phage SPN3US (**B**), and phage SPN10H (**C**). The amino acid sequences of a terminase large subunit were obtained from the NCBI database and aligned using MUSCLE. The phylogenetic trees were generated with MEGA 11.0. The numbers at the branch nodes indicate the bootstrap value (%) built on 1000 replications.

The phylogenetic analysis of the three phages, based on their large subunit of terminase, was conducted to elucidate the evolutionary relationships at the DNA level. SPN3UB aligns closely with enterobacteria phage ES18, a lysogenic phage targeting *Salmonella*, suggesting a potential lysogenic cycle in SPN3UB, as indicated by the presence of lysogeny control proteins and an integrase (Figure 3B; [19]). SPN3US shows the closest homology to phage SaP7, a polyvalent phage infecting both *Salmonella* and *Esherichia coli* (Figure 3A; [20]). SPN10H shares a close relationship with the *Salmonella* phage Stitch and Seabear, T5-like phages that are capable of infecting both *Salmonella* and *E. coli* (Figure 3C; [21,22,23]). Given SPN10H’s significant genomic resemblance to T5-like phages and its host range, including *Salmonella* and *E. coli* (Table 1), it is likely to be classified as a T5-like phage.

### 2.2. The Determination of the Host Range of the Salmonella-Targeting Phages

The host range of the three phages was determined against a total of 42 bacterial strains, including 38 *Salmonella* strains (comprising *S. typhimurium* and *S. enteritidis*), 4 other Gram-negative bacterial strains, and one Gram-positive strain, *Bacillus cereus*. SPN3UB created clear plaques against 17 out of 27 *S. typhimurium* strains and one *S. enteritidis* isolate strain. SPN3US was able to effectively kill 15 *S. typhimurium* strains and 3 *S. enteritidis* strains, displaying an expanded host range beyond that of SPN3UB. SPN10H showed a relatively broad host range, infecting 15 *S. typhimurium*, 9 *S. enteritidis* and 2 *E. coli* strains. In conclusion, the host range of each phage covered approximately 90% of the tested *Salmonella* strains. These results suggest the potential use of SPN3US, SPN3UB, and SPN10H as phage cocktail components to control *Salmonella* infections (Table 1).

### 2.3. Bacterial Challenge Assay

**Figure 4:**
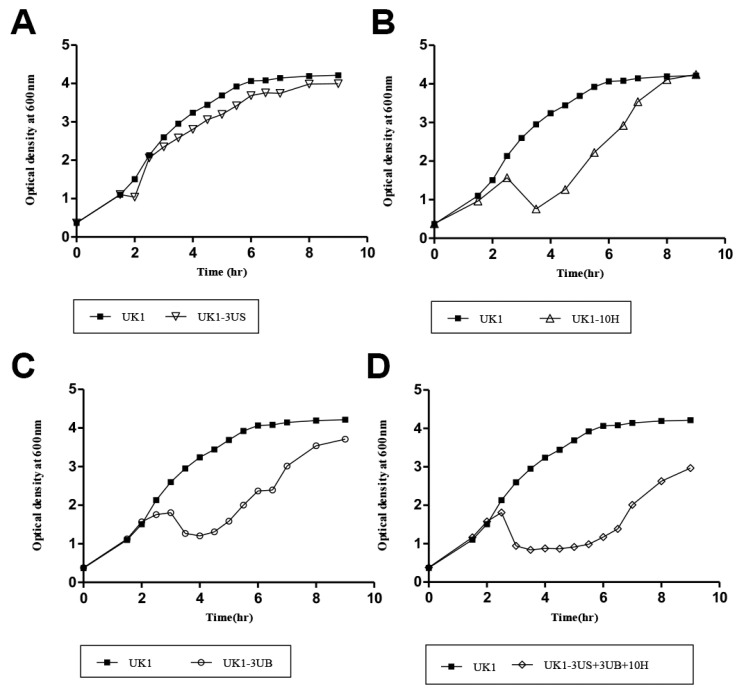
Bacterial challenge test results for the phages SPN3US (**A**), SPN10H (**B**), SPN3UB (**C**), and a phage cocktail (**D**) against *S. typhimurium* UK1. A representative graph was shown from 10 biological replicates, which displays the optical density (OD) at 600 nm, monitored every 30 min. *S. typhimurium* UK1 was challenged with each phage or the phage cocktail when the OD at 600 nm reached 0.5.

To test the lytic activity of each phage against *S. typhimurium* UK1, a strain effectively infected by all three phages, we challenged the host strain with SPN3UB, SPN3US, and SPN10H at an MOI of 1. The lysis of the bacterial host was evaluated by measuring the optical density at 600 nm at indicated time points. SPN3US caused only slight growth retardation at about 30 min rather than complete growth inhibition, after which SPN3US-infected *S. typhimurium* UK1 grew similarly to the uninfected control (Figure 4A). A significant decrease in OD_600_ was observed at about 4 h post-infection with SPN3UB or SPN10H, indicating their effective bacteria lysis (Figure 4B,C). However, this growth inhibition was maintained for only 1 h or less before the bacteria resumed growth, suggesting the emergence of phage-resistant bacteria. The three phages were formulated into a phage cocktail to control *S. typhimurium*. Despite SPN3UB being predicted to be a temperate phage, it was included in the phage cocktail due to its strong lytic activity and broad host range. While strictly lytic phages are generally preferred for therapeutic purposes, some studies have explored the potential of temperate phages, with some being engineered to remove unwanted genes for therapeutic use [30]. The treatment with phage cocktail inhibited the growth of *S. typhimurium* UK1 for 4 h, demonstrating a four times greater efficacy in delaying the emergence of resistance compared to single-phage treatment (Figure 4D). These results suggest that the phage cocktail can inhibit host bacterial growth more effectively and delay the emergence of phage resistance compared to a single phage infection. Previous studies have shown that the simultaneous use of several different phages, targeting different host surface receptors, can effectively suppress the development of anti-phage pathogens [31,32,33]. Notably, SPN3UB, SPN3US, and SPN10H target distinct surface receptors, including O-antigens of lipopolysaccharides [17], flagella [18] and BtuB [34], respectively. We reasoned that this superior efficacy of our phage cocktail in delaying the emergence of phage-resistant bacteria arises from the simultaneous targeting of varied host receptors, unlike single-phage use.

### 2.4. The Application of the Phage Cocktail to Prevent Salmonella Contamination in Foods

**Figure 5:**
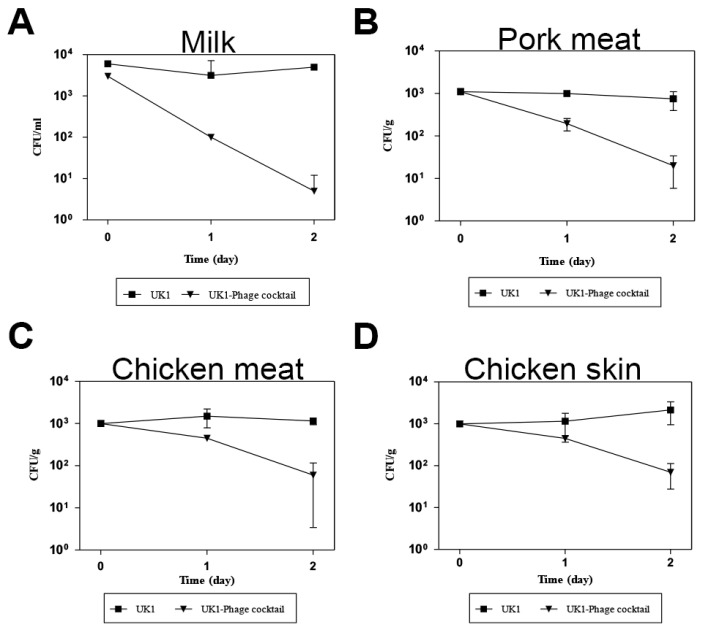
The evaluation of a phage cocktail’s efficacy in reducing *Salmonella* contamination across various food matrices. The assay was conducted in milk (**A**), pork meat (**B**), chicken tenders (**C**), and chicken skin (**D**) at 4 °C for 2 days.

Given that the phage cocktail containing SPN3US, SPN3UB, and SPN10H effectively controlled *Salmonella* bacterial growth in vitro (Figure 4D), we aimed to evaluate its lytic activity in pasteurized milk, pork meat, chicken meat, and chicken skin, which are commonly contaminated by *Salmonella*. To this end, these foods were artificially inoculated with *S. typhimurium* UK1 and treated with the phage cocktail. The growth inhibition was monitored at 4 °C because dairy or meat products are still at risk of *Salmonella* contamination even when refrigerated. The results demonstrated a significant 3-log reduction in bacterial growth in milk after a 2-day incubation (Figure 5A). However, a lesser reduction was observed on raw pork, chicken tender, and chicken skin, with decreases of 1.5 log CFU/g, 1 log CFU/g, and 1.4 log CFU/g, respectively (Figure 5B–D). These results are consistent with previously reported studies that phages exhibited stronger host bacterial reductions in liquid foods compared to solid or semi-solid food matrices [35,36,37] as phages are allowed to be diffused in liquid, making them more accessible to bacterial populations.

It is generally known that storing foods at cold temperatures, ranging from 5 °C to 8 °C, is beneficial as it can effectively inhibit *Salmonella* growth by suppressing its metabolic and enzymatic activities [38,39]. These physiological changes can limit the lytic activity of phages against the host by prolonging the proliferation and latent period, thus diminishing their replication rate and progeny production [40,41]. In light of this, a phage cocktail in this study is a promising biocontrol agent as it can effectively control *Salmonella* contamination at cold temperatures. Taken together, our findings suggest that the phage cocktail may effectively reduce *Salmonella* during cold storage of foods, and it could potentially serve as an alternative to antibiotics for controlling *Salmonella* contamination in various foods (Figure 5).

## 3. Materials and Methods

### 3.1. Bacterial Strains and Growth Condition

A total of 43 bacterial strains including *Salmonella*, *E. coli*, and *Cronobacter* strains used in this study are listed in Table 1. All bacterial strains were aerobically grown in Luria–Bertani medium (LB) broth (Difco, Detroit, MI, USA) at 37 °C.

### 3.2. Bacteriophage Isolation and Propagation

Phages were isolated from the sewage, feces, and litter samples collected in traditional markets and poultry farms (Seoul and Chuncheon, Republic of Korea). The samples were mixed with sterile Butterfield’s phosphate-buffered dilution water (0.25 M KH_2_PO_4_, pH 7.2) and homogenized using a blender (BacMixer 400; Interscience Laboratory Inc., St. Nom, France). The mixture was centrifuged at 9000× *g* for 10 min at 4 °C and the supernatant was filtered using a 0.22 µm polyethersulfone (PES) membrane filter (Millipore, Billerica, MA, USA) to remove bacterial cells. Then, 25 mL of the filtrate was mixed with an equal volume of LB broth and incubated for 12 h at 37 °C. Following centrifugation (9000× *g*, 10min) and filtration, 10 mL of the filtrate was mixed with 40 mL of LB broth and *S. typhimurium* SL1344, and the mixture was incubated at 37 °C for 12–18 h with shaking (220 rpm). The culture was centrifuged, and the supernatant was filtered as above. Phage plaques were confirmed by spotting the filtrate on 0.4% LB agar (soft agar) containing *S. typhimurium* SL1344. The agar plates were incubated overnight at 37 °C and monitored for plaque formation. Each single plaque was picked with a sterile tip and eluted in sterilized sodium chloride–magnesium sulfate (SM) buffer (50 mM Tris-HCl, pH 7.5, 100 mM NaCl, 10 mM MgSO_4_·7H_2_O). This plaque purification step was repeated at least three times. Finally, we purified 3 phages and named them: SPN3US, SPN3UB, and SPN10H.

For phage propagation, the phage lysate was added to the prophage-cured *S. typhimurium* strain LT2 [referred to as LT2(c)] that is exponentially grown at a multiplicity of infection (MOI) of 1, followed by incubation at 37 °C with shaking for 3–4 h. Cell debris was removed by subsequent centrifugation and filtration using 0.22 μm pore size filters, and phage particles were precipitated with polyethylene glycol (PEG) 6000 (Sigma, St. Louis, MO, USA). Finally, the propagated phages were concentrated by cesium chloride (CsCl) density gradient ultracentrifugation (78,500× *g* for 2 h at 4 °C). Viral particles were recovered and dialyzed with SM buffer stirring for 1 h at 4 °C.

### 3.3. Bacteriophage Host Range

The bacterial strains listed in Table 1 were incubated overnight at 37 °C. A 100 μL of each bacterial culture was mixed with 6 mL of soft agar and overlaid on LB agar plates. Subsequently, 10 μL of serially diluted phage lysates was spotted onto host bacterial lawns and incubated at 37 °C overnight. After incubation, the infectivity was determined based on the appearance of the spots: “C”, clear single plaques; “T”, turbid single plaques; “I”, inhibited growth without single plaques; “—”, no lysis.

### 3.4. Morphological Analysis by TEM

The three purified phages were subjected to TEM analysis to characterize their morphology. Briefly, 5 μL of high-titer phage stock (1 × 10^10^ plaque-forming units (PFU)/mL) was placed on carbon-coated copper grids and negatively stained with 2% aqueous uranyl acetate (pH 4.0). The samples were examined with a TEM (LIBRA 120, Carl Zeiss, Jena, Germany) at an 80 kV accelerating voltage at the National Institute of Agricultural Science and Technology (Suwon, Republic of Korea). The phages were morphologically classified according to the guidelines of the International Committee on Taxonomy of Viruses [42].

### 3.5. Phage DNA Extraction

Phage DNA was extracted by the phenol–chloroform method as previously described [43]. Briefly, phage lysate (10^9^ PFU/mL) was treated with RNaseA and DNase for 1 h at 37 °C to remove bacterial DNA and RNA contaminants. To degrade the phage capsid, phage lysates were then treated with lysis buffer containing 0.5 mol/L ethylene–diamine–tetraacetic acid (EDTA), 10 mg/mL proteinase K, and 1% sodium dodecyl sulfate (SDS) for 2 h at 56 °C.

### 3.6. Whole-Genome Sequencing and Genomic Analysis

The purified phage DNA was sequenced using a Genome Sequencer FLX (GS-FLX) Titanium sequencer (Roche, Mannheim, Germany) and assembled with 454 Newbler 2.3 assembler (Roche) at Macrogen Inc., Seoul, Republic of Korea. The open reading frames (ORFs) were identified with the ORF Finder at the National Center of Bioinformatics site (http://www.ncbi.nlm.nih.gov/gorf, accessed on 20 August 2011) and GenMark.hmm prokaryotic version 2.4 (http://opal.biology.gatech.edu/GeneMark/gmhmm2_prok.cgi, accessed on 20 August 2011). Sequence manipulations and genomic analysis were performed using CLC Genomics work-bench version 3.6.1 on a workstation at the Biopolymer Research Center for Advanced Materials, Sejong University. Phylogenetic analysis of amino acid sequences from the large subunit of the phage terminase was performed. The distances among the phages were determined by aligning their sequences using MUSCLE [44] and visualized using MEGA11 with 1000 bootstrap replicates, based on the neighbor-joining method [45,46].

### 3.7. Bacterial Challenge Assay

An exponentially grown *S. typhimurium* UK1 culture (optical density at 600 nm = 0.5) was infected with each phage or phage cocktail at an MOI of 1 and the optical density was monitored at 600 nm every 30 min with an uninfected culture as a negative control.

### 3.8. Biocontrol of Bacteria in Foods

Milk, Pork, chicken tender, and skin were purchased from the same retail store. First, milk was inoculated with *S. typhimurium* UK1 cells (~10^3^ CFU diluted from an overnight culture), then incubated at 5 °C after the addition of phage cocktail (~10^7^ PFU). The viable cells were counted at each time point by plating each sample on xylose lysine deoxycholate citrate (XLD) agar. Other food samples (pork, chicken tender, and chicken skin) were aseptically cut into 2 cm × 2 cm pieces in petri dishes. Subsequently, 20 μL of *S. typhimurium* UK1 cells (~10^3^ CFU) was spotted onto the surface of the samples and dried for 10 min at room temperature for bacterial attachment to the samples. Then, 200 μL of prepared phage cocktail was added to cover the entire surface of the food samples and incubated at 5 °C. The intact food samples were treated with SM buffer without the phage cocktail and incubated in parallel as a negative control. At the indicated time points, each sample was harvested and homogenized with SM buffer in a stomacher to detach bacterial cells from the samples. The homogenized samples were then serially diluted and plated on the XLD agar to enumerate viable *Salmonella*. All experiments were conducted in duplicate.

## 4. Conclusions

We evaluated the efficacy of a phage cocktail comprising SPN3US, SPN3UB, and SPN10H as a novel biocontrol strategy against *Salmonella* enterica in various food products. Through morphological and genomic analyses, these phages were identified as members of the *Caudoviricetes* class. Each phage targets a distinct bacterial surface component as receptors, which broadens their antimicrobial spectrum. The phage cocktail exhibited significant lytic activity against *S. typhimurium* UK1, effectively delaying the emergence of phage-resistant bacteria. Our findings demonstrate that phage treatment effectively reduces *Salmonella* levels on chicken meat/skin and in milk at a refrigerated temperature. This suggests that phages can be used to inhibit cross-contamination by *Salmonella* and serve as an antimicrobial agent during the cold storage of foods, which is a crucial factor in food distribution. Considering that the phage cocktail can effectively target both *S. typhimurium* and *S*. Enteriditis, the most prevalent serovars in poultry [47], our phage cocktail could be applied to the poultry industry. Overall, it could potentially serve as an effective and sustainable alternative to antibiotics for controlling *Salmonella* contamination in various food industries.

## Figures and Tables

**Table 1:** Host range of the phages SPN3UB, SPN3US, and SPN10H.

Host Strains		Lytic Activity of Phage ^1^			
		SPN3UB	SPN3US	SPN10H	Source or Reference ^2^
*S. typhimurium*	LT2	C	C	C	[24]
	UK1	C	C	C	[25]
	SL1344	C	C	C	NCTC
	14028S	I	C	I	ATCC
	DT104	C	C	I	[26]
	ATCC 19586	C	C	C	ATCC
	ATCC 43174	C	C	I	ATCC
	3068	C	C	I	Laboratory collection
	ATCC 12023	C	C	C	ATCC
	BJ 3505	-	C	C	Laboratory collection
	CS 634	I	I	T	Laboratory collection
	CS 800	I	C	C	Laboratory collection
	KCTC 1425	-	C	C	KCTC
	KCTC 1925	-	I	C	KCTC
	S.T 4174	C	C	C	Laboratory collection
	ST DB7155	C	C	C	[27]
	NCTC 12023	I	C	C	NCTC
	Isolate 1	I	-	-	Laboratory collection
	Isolate 2	C	-	-	Laboratory collection
	Isolate 3	C	-	I	Laboratory collection
	Isolate 4	I	-	T	Laboratory collection
	Isolate 5	C	-	I	Laboratory collection
	Isolate 6	C	C	C	Laboratory collection
	Isolate 7	C	-	I	Laboratory collection
	Isolate 8	-	I	I	Laboratory collection
	Isolate 9	C	C	C	Laboratory collection
	Isolate 10	C	-	C	Laboratory collection
*S. enteritidis*	ATCC 13076	-	I	C	Laboratory collection
	Isolate 1	-	I	C	Laboratory collection
	Isolate 2	-	C	C	Laboratory collection
	Isolate 3	-	I	T	Laboratory collection
	Isolate 4	C	-	T	Laboratory collection
	Isolate 5	-	C	C	Laboratory collection
	Isolate 6	I	I	C	Laboratory collection
	Isolate 7	I	I	C	Laboratory collection
	Isolate 8	-	C	C	Laboratory collection
	Isolate 9	-	I	C	Laboratory collection
	Isolate 10	I	I	C	Laboratory collection
Other Gram-negative bacteria	*E. coli* MG1655	-	-	C	[28]
	*E. coli* DH5a	-	-	C	[29]
	*E. coli* O157:H7 ATCC 35150	-	-	I	ATCC
	*Cronobacter sakazakii* ATCC 29544	-	I	-	ATCC
Gram-positive bacteria	*B. cereus* NRRL B-569	-	-	-	NCTC

^1^ C, clear single plaques; T, turbid single plaques; I, inhibited growth without single plaques; -, no lysis. ^2^ ATCC, American Type Culture Collection; NCTC, National Collection of Type Cultures; KCTC, Korean Collection of Type Cultures.

## Data Availability

The original contributions presented in the study are included in the article; further inquiries can be directed to the corresponding authors.
